# Multiple imputation for estimating hazard ratios and predictive abilities in case-cohort surveys

**DOI:** 10.1186/1471-2288-12-24

**Published:** 2012-03-09

**Authors:** Helena Marti, Laure Carcaillon, Michel Chavance

**Affiliations:** 1 Inserm, CESP Centre for Research in Epidemiology and Population Health, U1018, Biostatistics team, F-94807 Villejuif, France; 2 Inserm, CESP Centre for Research in Epidemiology and Population Health, U1018, Hormones and Cardiovascular Disease team, F-94807 Villejuif, France

## Abstract

**Background:**

The weighted estimators generally used for analyzing case-cohort studies are not fully efficient and naive estimates of the predictive ability of a model from case-cohort data depend on the subcohort size. However, case-cohort studies represent a special type of incomplete data, and methods for analyzing incomplete data should be appropriate, in particular multiple imputation (MI).

**Methods:**

We performed simulations to validate the MI approach for estimating hazard ratios and the predictive ability of a model or of an additional variable in case-cohort surveys. As an illustration, we analyzed a case-cohort survey from the Three-City study to estimate the predictive ability of D-dimer plasma concentration on coronary heart disease (CHD) and on vascular dementia (VaD) risks.

**Results:**

When the imputation model of the phase-2 variable was correctly specified, MI estimates of hazard ratios and predictive abilities were similar to those obtained with full data. When the imputation model was misspecified, MI could provide biased estimates of hazard ratios and predictive abilities. In the Three-City case-cohort study, elevated D-dimer levels increased the risk of VaD (hazard ratio for two consecutive tertiles = 1.69, 95%CI: 1.63-1.74). However, D-dimer levels did not improve the predictive ability of the model.

**Conclusions:**

MI is a simple approach for analyzing case-cohort data and provides an easy evaluation of the predictive ability of a model or of an additional variable.

## Background

Case-cohort surveys produce incomplete data by design. A subcohort is selected by simple or stratified random sampling, all subjects are followed up and the events of interest are recorded. The phase-1 variables are observed for the entire cohort, whilethe phase-2 variables are only known for the case-cohort sample, i.e., subjects belonging to the subcohort and all those presenting the event of interest [1]. Thus, in case-cohort studies, the non-cases who do not belong to the subcohort are incompletely observed by design, enabling cost reduction with a small loss of efficiency.

Various approaches have been described to estimate the proportional hazard model in case-cohort surveys: Weighted estimators [2-6] are classically used in these surveys, with analysis restricted to the completely observed subsample, so the information collected for incompletely observed non-cases is ignored and inefficient estimators for the effect of phase-1 variables are obtained. One of the most popular is the Borgan II estimator [4]. Scheike and Martinussen [7] proposed a maximum likelihood estimator based on proportional hazards assumption, using the EM algorithm [8], therebyincreasing efficiency as compared to weighted estimators when the relative risk and disease incidence are high. However, in general, the studied disease incidence in case-cohort surveys is low. Breslow *et al. *[9] suggested calibrating or estimating the weights a posteriori, using all the phase-1 information, to improve precision with respect to classical weighted estimators. Marti and Chavance [10] showed that multiple imputation (MI) is a good alternative to classical weighted methods for the analysis of case-cohort data. When the imputation model was correct, the MI approach provided unbiased estimators of the log hazard ratios and correctly estimated the variance of its estimators. As expected, the MI approach was more precise than the usual weighted estimators for the parameters associated with phase-1 variables. The former was also slightly more precise than the latter for the phase-2 variable. In Marti and Chavance [10] the imputations were performed according to a correctly specified imputation model. However, in practise, the distribution of the phase-2 variable is unknown and onemay wonder how MI compares to weighted estimators when the imputation model is misspecified.

No standard method exists for quantifying the usefulness or predictive ability of a model or an additional variable in the framework of case-cohort surveys. The predictive ability can be measured in terms of calibration, which refers to the ability of a model to match predicted and observed values, when we are interested in individual predictions; or in terms of discrimination, which refers to the ability of a model to distinguish between subjects with or without a binary event, when we are interested in identifying a group of high-risk subjects. In the present work, we focus on discrimination.

As shown below, a naive measurement of predictive ability from case-cohort data often leads to a biased estimate of the predictive ability because it varies with the censoring rate and thus depends on the subcohort size. Alternatively, because MI reconstitutes whole cohorts, any tool developed to estimate the predictive ability in the framework of cohort surveys can be applied to case-cohort data, so we propose using the MI approach to estimate the predictive ability ofa model or of an additional variable and their standard errors.

The objectives of this study were 1) to evaluate MI for estimating hazard ratios when the distribution of the phase-2 variable is misspecified; and 2) to present an adequate methodology for estimating the predictive ability of a model or of an additional variable in case-cohort surveys. We performed a simulation study to validate the MI approach for estimating the predictive ability of a model or of an additional variable and to assess its potential limits. As an illustration, we analyzed case-cohort data from the Three-City study [11] to estimate the predictive ability of the D-dimer plasma concentration, a marker of coagulation and fibrinolysis, on coronary heart disease (CHD) and on vascular dementia (VaD) risks.

## Methods

### Incomplete observations and multiple imputation

Case-cohort surveys are a particular type of incomplete observations, in which data are missing at random [12] by design, as the probability of being completely observed depends only on the case status, with simple random sampling, and on some phase-1 variables with stratified sampling. MI is a simple and efficient method for analyzing incomplete observations, while taking into account all the levels of uncertainty regarding missing values. This provides an approximation of the maximumlikelihood estimator and thus enables the potential selection bias to be corrected. This method relies on the generation of several plausibly completed data sets (*M *≥ 2), accounting for all levels of uncertainty concerning the missing values. A prediction model must be built, taking into consideration the relationships between the incomplete variable and the other variables, as observed in the complete part of the data. The missing data are not replaced by their expectation but by a value drawnfrom the distribution posited by the model. To take into account the uncertainty concerning the parameters of the imputation model, several imputations are performed with parameters drawn from the asymptotic distribution of their estimator. An estimate of the parameter of interest, ${\hat{\theta }}_{m},m=\left\{1,\ldots ,M\right\},$ and an estimate of the variance of the estimator, $\hat{V}({\hat{\theta }}_{m}),$ are obtained from each completed data set. If the imputation model is correct, these estimators are not biased. The MI estimate, also unbiased, is the mean of the *M *estimates:

(1) $${\hat{\theta }}_{MI}=\frac{1}{M}\overset{M}{\underset{m=1}{\sum }}{\hat{\theta }}_{m}$$

where *M *is the number of completed data sets and ${\hat{\theta }}_{m}\text{,}\;m\text{ = \{ 1,}\ldots \text{,}M\text{\}}$ is the estimate of the parameter of interest provided by the *m^th ^*completed data set. The multiplicity of imputations enables correct estimation of the variance of this single estimator, which is the sum of 2 components: the within-imputations component, *W_MI_*, and the between-imputations component, *B_MI_*:

(2) $$\begin{array}{ccc} \hat{V}\left({\hat{\theta }}_{MI}\right) & ={Ŵ}_{MI}+{\hat{B}}_{MI} & \\ & =\frac{1}{M}\overset{M}{\underset{m=1}{\sum }}\hat{V}\left({\hat{\theta }}_{m}\right)+\left(1+M^{-1}\right)\frac{\sum_{m=1}^{M}\left({\hat{\theta }}_{m}-{\hat{\theta }}_{MI}\right){\left({\hat{\theta }}_{m}-{\hat{\theta }}_{MI}\right)}^{\prime }}{M-1} \end{array}$$

where the factor 1 + *M*^-1 ^is an adjustment for using a finite number of imputations [13].

MI requires a model correctly reflecting the relationship between the incomplete variable and the outcome of interest. In case-cohort surveys, we need to impute phase-2 variable values for the non-cases who do not belong to the subcohort. Under the rare disease assumption, we have shown that a simple generalized linear model, using all the complete data (cases and non-cases) and including the case indicator among the explanatory variables, has to be considered [10]. Practically, in addition to the case indicator and the stratification variables, when the subcohort was selected by stratified sampling, it is necessary to include in the imputation model all the variables appearing in the proportional hazard model. Because imputations are based on asymptotic distributions, caution is necessary, since if too few subjects present the event of interest, the distribution of the estimators can differ from the asymptotic one. As a consequence, the maximum likelihood estimator of the imputation model could be biased or not normally distributed.

### Predictive ability of a model and of a supplementary variable

Harrell *et al. *[14] proposed the *C *index to measure the predictive ability of a model in cohort studies as the agreement between the order of the predicted and observed survival times in any pair of subjects (the event of interest is assumed to be death, leading to the use of survival terminology). That is, the concordance probability using all pairs of subjects in the population. However, with censored data, it is not possible to consider all the pairs of subjects because survival time is not observed for censored subjects. Let *T_i _*be the survival time for subject *i*, *i *= 1,...,*N*, where *N *is the cohort size, and *C_i _*the censoring time for subject *i*. We observe *X_i _*= *min*(*T_i_*, *C_i_*). Usable pairs are those for which the order of the predicted survival times can be compared to the order of the true survival times, i.e., pairs formed by 2 uncensored subjects or an uncensored subject and a subject censored after the uncensored subject's death. A pair of censored subjects carries no information about its agreement with the expected survival provided by the model since the order of the survival times is not known. Similarly a pair formed by a subject whose survival time is observed and a subject censored before this survival time provides no information on this agreement since the unknown survival time could be anterior or posterior to the observed one. Harrell *et al. *[15] showed that, in the common models used for survival analysis, such as the proportional hazard model, the predicted survival times and the predicted survival probabilities at a fixed time *t *can be interchanged for the comparison. The Harrell's *C *index is defined as:

(3) $$C=\frac{\pi_{c}}{\pi_{c}+\pi_{d}}$$

where π*_c _*is the probability of concordance for a pair *(i,j) *and π*_d _*is the probability of discordance. We assume continuous survival times and continuous predicted survival probabilities, so *P*(*X_i _*= *X_j_*) = *P*(*Y_i _*= *Y_j_*) = 0, thus *π_c _*+ *π_d _*= 1. *C *is estimated by the proportion of concordant pairs among the usable pairs. The estimated variance was given by Kremers [16].

In practice, we are often interested in estimating the predictive ability of an additional phase-2 variable. Let *M*_1 _be a proportional hazard model including only phase-1 variables, and *C*_1 _and *SE*_C1 _respectively the *C *indexof *M*_1 _and its standard error. Let *M*_2 _be a proportional hazard model adding the phase-2 variable to *M*_1_, and *C*_2 _and *SE*_*C*2_, respectively, the *C *index of *M*_2 _and its standard error. Harrell's predictive ability ofthe added phase-2 variable is Δ = *C*_2 _- *C*_1 _. Complementary measures of predictive ability of a new variable, such as the net reclassification improvement (NRI) and the integrated discrimination index (IDI), were proposed by Pencina [17]. NRI needs some a priori meaningful risk categories. It quantifies the correct reclassification introduced by using a model with the added variable as compared to the classification obtained without this variable. The IDI can be viewed as a continuous version of the NRI with probabilities used instead of categories. It can be defined as the discrimination-slope difference between the models with and without a quantitative variable. To estimate the predictive ability of a model or of an additional variable, we reconstructed plausible whole cohorts using MI. For each reconstructed whole cohort, we could then directly obtain *C*_1_, *SE*_*C*1_, *C*_2_, *SE*_*C*2_, Δ, NRI, IDI and their respective variances. Using equations (1) and (2), we obtained the MI estimates of these quantities. Concerning the variance of Δ, the between-imputation component is estimated by the empirical variance of the *M *estimates of Δ provided by the *M *completed data sets. However, for the within-imputation component, the asymptotic variance of the estimator provided by a complete data set, does not have an analytical form. With a fully observed cohort, bootstrapping is a way to estimate the variance of the corresponding Δ. Therefore, each whole cohort reconstructed by MI has to be resampled. In the simulations as in the real data analysis, we used 100 bootstrap samples.

### Simulation study

Two phase-1 variables were simulated: a binary variable, *Z*_1_, and a Gaussian variable, *Z*_3_, observed for the entire cohort. For the phase-2 variable, *Z*_2_, we considered three different distributions: normal, log-normal and uniform, all of them with unit variance, independent of *Z*_1_, but having a correlation coefficient of 0.2 with *Z*_3_. The survival time had an exponential distribution, with *λ *= *exp *(*β*_1_*Z*_1 _+ *β*_2_*Z*_2 _+ *β*_3_*Z*_3 _). *β*_1_, *β*_2 _and *β*_3 _were fixed at the same value and set at 0 or log(2). The censoring time followed a uniform distribution over the interval [0,τ], where *τ *was chosen so that the probability of an event was approximately 0.03 (*τ*= 0.025). The cohort size was 10,000. We also simulated a phase-1 variable predictive of *Z*_2_, ${\tilde{Z}}_{2}\equiv Z_{2}+\varepsilon$with *ε ~ N *(0, *σ*^2^) independent of *Z*_2_. The variance σ^2 ^was fixed at 1 which corresponds to a correlation between $Z_{2}\;\text{and}\;{\tilde{Z}}_{2}$ of approximately 0.7. We wanted to estimate the effect of *Z*_2 _on survival time and its predictive ability. The cohort was divided into 9 strata based on the tertiles of ${\tilde{Z}}_{2}\;\text{and}\;Z_{3}$, and the non-cases were chosen by stratified sampling. Case-cohort sampling was simulated with 1,000 subjects in each subcohort. The phase-2 variable was not available for non-cases not included in the subcohort, so MI was used to complete the data set. Thus, we built the same linear prediction model for each *Z*_2 _based on the stratification phase-1 variable and the case indicator. *Z*_3 _was not directly included in the imputation model to predict *Z*_2_, because it was a stratification variable included in the model and because of the weak correlation between *Z*_2 _and *Z*_3_. The imputation model was: *Z*_2 _= *α*_0 _+ *α*_1 _*I_case _*+ *α*_2 _*Strata *+ *ε *, where *α*_0 _and *α*_1 _are scalar, *α*_2 _is a vector coefficient, *I_case _*is the case indicator, *Strata *is the vector of stratum indicators and *є *is the vector of errors independently and identically distributed ~ *N *(0, *σ*). Thus, the imputation model was correctly specified for *Z*_2 _normally distributed but misspecified for *Z*_2 _log-normally or uniformly distributed. One thousand cohorts were simulated for each scenario.

Five imputations were performed and 5 complete data sets were generated for each cohort. We estimated the log hazard ratios using MI and the "Borgan II" weighted estimator [4]. We used MI to estimate the predictive ability of models with and without the phase-2 variable, and the predictive ability of the phase-2 variable, NRI and IDI. We also studied the consistency of the naive estimator of Harrell's C index in case-cohort surveys by varying the subcohort size. Using the above simulation conditions and, exceptionally, a scenario with *β_1 _*= *β_3 _*= *log*(2) and *β_2 _*= *log*(1.5), we simulated case-cohort samples with the subcohort size set at 300 or 1,000 subjects. We estimated the predictive ability in the case-cohort samples and in the multiply imputed data sets.

### Case-cohort survey from Three-City study

Briefly, the 3C-Study was designed to examine the relationship between vascular diseases and dementia in a community housing 9,294 persons aged 65 years and over between 1999 and 2001 in three French cities. The detailed methodology has been previously described [11]. A case-cohort substudy was conducted [18], to investigate the relationship between biomarkers, such as plasma levels of D-dimer (a marker of coagulation and fibrinolysis) and the 4-year incidence of coronary heart disease (CHD), stroke and all subtypes of dementia, including vascular dementia (VaD), in an elderly population. The phase-1 variables provided information on socio-demographic characteristics, education, medical history, diet, alcohol and tobacco use. Blood pressure, height and weight were also available. A subcohort of size n = 1,254, (13.5% of the full cohort) was randomly selected, stratifying on age, sex and recruitment center. Observed cumulated incidences of CHD and VaD were approximately 2% and 0.6%, respectively. Plasma D-dimer levels were only available for phase-2 subjects. Carcaillon *et al. *[18] treated quintiles of D-dimer level both qualitatively and linearly. They reported a linear increase in the risk of VaD according to D-dimer quintiles.

We re-assessed the relationship between plasma D-dimer levels and the risk of CHD and VaD, using MI and weighted estimators, and evaluated the predictive ability of D-dimer levels on both risks. We included the same explanatory variables as Carcaillon *et al. *[18] although we used tertiles of D-dimer rather than quintiles, to estimate CHD and VaD risks, due to the small number of events. Therefore, to estimate the risk of CHD, the proportional hazard model includedthe phase-1 variables: age, sex, center, body mass index, hypertension, hypercholesterolemia, diabetes, tobacco use, diabetes drugs, and as phase-2 variables, indicators of D-dimer tertiles. To estimate the risk of VaD, the proportional hazard model included the phase-1 variables: age, sex, centre, educational level, body mass index, the presence or absence of an apolipoprotein є4 allele and indicators of D-dimer tertiles.

For each outcome (CHD or VaD), it was necessary to reproduce the relationships among the incomplete variable, the outcomes and the confounder variables. For each outcome, we built an imputation model of tertiles of D-dimer levels, including the variables used in the proportional hazard model and the case-indicator. We estimated the predictive ability of proportional hazard models, without (*C*_1_) and with (*C*_2_) D-dimer levels, Δ = *C*2 - *C*1, and IDI for CHD and VaD risks. The NRI requires that some a priori meaningful risk categories be known. Based on the Third Adult Treatment Panel [ATP III] [19] risk classification for the 10-year risk of CHD, we adapted the cut-offs to 4-year risk. For VaD, we do not know a priori meaningful risk categories and did not compute NRI.

## Results

### Simulation study

The mean fraction of missing information about the effect of *Z*_2 _ranged from 5 to 14 percent when *β*_2 _= 0 and from 23 to 30 per cent when *β*_2 _= *log*(2) (data not shown). For each estimator (full cohort, case-cohort with MI and case-cohort with weights), we give the mean of the estimated coefficients, the mean of their standard error estimates, the observed standard error of the estimated coefficients and the mean squared errors of 1000 simulations (Table 1). Not surprisingly, the full cohort estimates and the case-cohort weighted estimates of the log hazard ratios were unbiased. Similarly, with a correctly specified normal imputation model, all MI estimates were unbiased. With a misspecified normal imputation model, MI estimate of the effect *β*_2 _= *log*(2) of *Z*_2 _was biased (-13%) when *Z*_2 _was log-normally distributed. When *Z*_2 _was uniformly distributed, MI estimate of the effect of *Z*_2 _was slightly biased (-5%). With a misspecified normal imputation model and *β*_2_= 0, no bias was observed. TheMI variance and the weighted estimator variance agreed with the observed dispersions of the estimates. The observed dispersion was always smaller with MI than with the weighted estimator. For the phase-1 variables, this dispersion was similar for the entire cohort and with MI, whatever the distribution of the phase-2 variable. For the estimated effect of the phase-2 variable, the observed standard deviations were smaller with MI than with the Borgan II weighted estimator but, as expected, slightly larger with MI than in the full cohort analyses. Altogether, the mean squared errors were smaller with MI than with the weighted estimator, except for the effect of the phase-2 variable with *β*_2 _= *log*(2) and *Z*_2 _was log-normally distributed.

**Table 1:** Mean of the log hazard ratio estimates (Est), mean of the standard error estimates $\overset{\wedge }{\mathrm{SE}}$, standard error of the estimates (SE) and mean of the mean square error (MSE). Results of 1,000 simulations.

		Full cohort				Multiple imputation *^a^*				Weighted estimator		
	
	Est	$\overset{\wedge }{\mathrm{SE}}$	SE	MSE	Est	$\overset{\wedge }{\mathrm{SE}}$	SE	MSE	Est	$\overset{\wedge }{\mathrm{SE}}$	SE	MSE
Z_2 _normally distributed												
*β*_1 _= *β*_2 _= *β*_3 _= 0												
*β*_1_	-0.003	0.107	0.100	0.010	-0.003	0.107	0.110	0.010	-0.001	0.133	0.128	0.016
*β*_2_	-0.001	0.054	0.058	0.003	-0.001	0.060	0.062	0.004	0.001	0.065	0.068	0.005
*β*3	-0.004	0.053	0.056	0.003	-0.004	0.054	0.057	0.003	-0.003	0.058	0.060	0.004
*β*_1 _= *β*_2 _= *β*_3 _= *log*(2)												
*β*_1_	0.689	0.118	0.113	0.013	0.676	0.119	0.112	0.013	0.696	0.168	0.165	0.027
*β*_2_	0.687	0.058	0.057	0.003	0.679	0.070	0.068	0.005	0.701	0.088	0.097	0.009
*β*_3_	0.683	0.057	0.057	0.003	0.679	0.058	0.058	0.004	0.689	0.080	0.090	0.007

Z_2 _log normally distributed												
*β*_1 _= *β*_2 _= *β*_3 _= 0												
*β*_1_	-0.003	0.107	0.100	0.010	-0.003	0.107	0.100	0.010	-0.004	0.133	0.128	0.016
*β*2	-0.001	0.027	0.034	0.001	0.015	0.031	0.032	0.001	0.002	0.034	0.038	0.001
*β*_3_	-0.004	0.053	0.056	0.003	-0.004	0.054	0.058	0.004	-0.005	0.059	0.062	0.004
*β*_1 _= *β*_2 _= *β*_3 _= *log*(2)												
*β*_1_	0.686	0.058	0.056	0.003	0.621	0.061	0.055	0.008	0.686	0.112	0.117	0.014
*β*_2_	0.692	0.013	0.015	2e0^-**4**^	0.602	0.015	0.014	0.008	0.695	0.020	0.023	0.001
*β*_3_	0.685	0.029	0.031	0.001	0.686	0.032	0.031	0.001	0.687	0.049	0.053	0.003

Z_2 _uniformly distributed												
*β*_1 _= *β*_2 _= *β*_3 _= 0												
*β*_1_	0.007	0.181	0.175	0.031	0.007	0.181	0.175	0.031	0.007	0.197	0.188	0.035
*β*_2_	-0.001	0.092	0.087	0.008	0.004	0.094	0.088	0.008	-0.002	0.098	0.095	0.009
*β*_3_	0.003	0.090	0.090	0.008	0.002	0.090	0.090	0.008	0.004	0.093	0.093	0.009
*β*_1 _= *β*_2 _= *β*_3 _= *log*(2)												
*β*_1_	0.690	0.120	0.116	0.013	0.680	0.121	0.115	0.013	0.694	0.166	0.169	0.028
*β*_2_	0.695	0.069	0.063	0.004	0.656	0.075	0.066	0.006	0.698	0.087	0.082	0.007
*β*_3_	0.690	0.058	0.054	0.003	0.689	0.059	0.055	0.003	0.698	0.081	0.081	0.007

^a ^MI estimates with imputation model: *Z*_2 _= *α*_0 _+ *α*_1_*Ind_case _*+ *α*_2_*Strata *+ *ε*, *ε *~ *N*(0, *σ*)

The results concerning the consistency of the naive estimator of Harrell's *C *index are reported in Table 2. In the scenario *β*_1 _= *β *_2 _= *β*_3 _= 0, the mean *C *index was nearly 0.5 for both models, without and with *Z*_2_, whatever the analysis performed. In the scenarios *β*_1 _= *β*_3 _= *log*(2) and *β*_2 _= *log*(1.5) or *β*_2 _= *log*(2), the naive computation of *C *with the case-cohort data led to lower predictive ability than with the full cohort, especially for the smaller subcohort. Bycontrast, the Harrell's *C *indexes estimated by MI were similar to those computed for the full cohort and did not depend on the subcohort size. The estimated dispersion of the *C *index was slightly greater than the observed dispersion of the estimates. The rejection percentage of the null hypothesis Δ = 0 was always similar to the full cohort analysis and to MI. As a consequence of the standard error overestimation, the observed first type error rate was slightly lower than 5%. Nevertheless, in the considered scenarios, the observed power was very high. As expected, the loss of power when comparing case-cohort with MI to full cohort analysis was small: with *β*_2 _= *log*(1.5), the observed power was 84.6% with a subsample size of 300, and 90.6% with a subsample size of 1000 versus 91.6% with the full cohort. MI estimates of NRI and IDI indexes were close to those obtained with the full cohort analysis and did not depend on the subcohort size. As compared to the full cohort results, the rejection percentage of the null hypothesis NRI = 0 was smaller with MI analysis when *β*_2 _= 0, larger when *β*_2 _= *log*(1.5) and similar when *β*_2 _= *log*(2). When the effect of the phase-2 variable was not null, the rejection percentage of the null hypothesis IDI = 0 was similar with MI and with full cohort analysis. By contrast, whatever the effect of the phase-2 variable, the estimation of NRI and IDI in the case-cohort sample provided larger measures of these indexes than the full cohort analysis.

**Table 2:** Mean of the predictive ability estimates (Est), mean of the standard error estimates $\overset{\wedge }{\mathrm{SE}}$ and standard error of the estimates (SE).

		*β_1 _*= *β_2 _*= *β_3 _= *0			*β_1 _*= *β_3 _*= *log*(2), *β_2 _*= *log*(1.5)				*β*1 = *β*2 = *β*3 = *log*(2)			
	
	Est	$\overset{\wedge }{\mathrm{SE}}$	SE	% *H*_0 _rejected	Est	$\overset{\wedge }{\mathrm{SE}}$	SE	% *H*_0 _rejected	Est	$\overset{\wedge }{\mathrm{SE}}$	SE	% *H*_0 _rejected
Full Cohort												
*C*_1_	0.518	0.033	0.012		0.727	0.032	0.015		0.733	0.029	0.014	
*C*_2_	0.524	0.033	0.013		0.747	0.031	0.015		0.733	0.029	0.014	
$\underset{}{\Delta }$	0.006	0.010	0.009	3.7	0.020	0.007	0.007	91.6	0.049	0.010	0.010	100
NRI	0.007	0.017	0.019	4.8	0.071	0.030	0.033	52.5	0.167	0.034	0.035	99.9
IDI	2e^-4^	2e^-4^	3e^-4^	6.0	0.014	0.003	0.005	99.9	0.048	0.006	0.009	99.9

MI1000												
*C*_1_	0.518	0.033	0.012		0.724	0.032	0.016		0.733	0.029	0.014	
*C*_2_	0.526	0.033	0.013		0.745	0.031	0.016		0.783	0.027	0.014	
Δ	0.008	0.012	0.010	3.4	0.021	0.008	0.008	90.6	0.049	0.010	0.011	100
NRI	0.009	0.019	0.017	1.5	0.076	0.033	0.033	64.8	0.172	0.037	0.036	100
IDI	3e^-4^	3e^-4^	4e^-4^	3.5	0.014	0.004	0.005	99.0	0.045	0.008	0.010	100

MI300												
*C*_1_	0.518	0.033	0.012		0.724	0.032	0.016		0.733	0.029	0.014	
*C*_2_	0.528	0.033	0.012		0.745	0.031	0.017		0.783	0.027	0.015	
Δ	0.010	0.014	0.011	3.0	0.021	0.008	0.009	84.6	0.050	0.011	0.012	100
NRI	0.013	0.023	0.018	1.3	0.076	0.035	0.035	57.0	0.172	0.039	0.039	99.7
IDI	4e^-4^	4e^-4^	5e^-4^	1.8	0.014	0.005	0.006	87.5	0.046	0.010	0.012	100

CC1000												
*C*_1_	0.528	0.032	0.013		0.667	0.033	0.015		0.670	0.031	0.014	
*C*_2_	0.534	0.033	0.015		0.709	0.032	0.022		0.737	0.029	0.014	
Δ	0.006	0.010	0.010	4.7	0.043	0.011	0.017	100	0.067	0.012	0.012	100
NRI	0.017	0.031	0.033	6.7	0.147	0.039	0.043	96.7	0.261	0.041	0.043	100
IDI	0.002	0.001	0.003	15.2	0.058	0.009	0.014	100	0.114	0.011	0.017	100

CC300												
*C*_1_	0.523	0.034	0.013		0.620	0.037	0.016		0.620	0.034	0.015	
*C*_2_	0.529	0.034	0.015		0.647	0.036	0.016		0.668	0.032	0.015	
Δ	0.006	0.010	0.009	3.6	0.027	0.011	0.011	83.3	0.048	0.013	0.013	99.8
NRI	0.019	0.039	0.043	6.2	0.154	0.043	0.050	94.4	0.257	0.046	0.051	99.9
IDI	0.002	0.001	0.003	13.9	0.040	0.008	0.014	99.8	0.078	0.010	0.017	100

Results from 1000 simulations

*C*_1 _Harrell's C index of the proportional hazard model without the phase-2 variable

*C*_2 _Harrell's C index of the proportional hazard model with the phase-2 variable

Δ, Harrell's predictive value of the phase-2 variable, *H*_0_: Δ = 0

NRI, Net reclassification index by adding the phase-2 variable, *H*_0_: NRI = 0 IDI, Integrated discrimination index by adding the phase-2 variable, H_0_: IDI = 0

Cohort, full cohort estimates; MI300, MI1000: multiple imputation estimates with subcohort sizes set, respectively, at 300 and 1,000; CC300, CC1000, case-cohort estimates with subcohort sizes set, respectively, at 300 and 1,000

Table 3 gives the results of the estimated predictive abilities for the correctly specified and the two misspecified normal imputation models. Full cohort analysis and MI provided similar predictive abilities estimates when the imputation model was correctly specified or when the phase-2 variable had no effect on the studied risk. In the scenario *β*_1 _= *β*_2 _= *β*_3 _= *log*(2), when *Z*_2 _was uniformly distributed, MI and full cohort analysis still provided similar estimates. However, when *Z*_2 _was log-normally distributed, the MI estimate was slightly smaller than the full cohort estimate -15%).

**Table 3:** Predictive ability of the two models and of the phase-2 variable.

		Full cohort				Multiple imputation		
	
	Est	$\overset{\wedge }{\mathrm{SE}}$	SE	% *H*_0 _rejected	Est	$\overset{\wedge }{\mathrm{SE}}$	SE	% *H*_0 _rejected
Z_2 _normally distributed								
*β*_1 _= *β*_2 _= *β*_3 _= 0								
*C*_1_	0.518	0.033	0.012		0.518	0.033	0.012	
*C*_2_	0.524	0.033	0.013		0.526	0.033	0.013	
Δ	0.006	0.010	0.010	3.7	0.008	0.012	0.010	3.4
*β*_1 _= *β*_2 _= *β*_3 _= *log*(2)								
*C*_1_	0.733	0.029	0.014		0.733	0.029	0.014	
*C*_2_	0.783	0.027	0.013		0.783	0.027	0.014	
Δ	0.049	0.010	0.010	100	0.049	0.010	0.011	100

Z_2 _normally distributed								
*β*_1 _= *β*_2 _= *β*_3 _= 0								
*C*_1_	0.518	0.033	0.012		0.518	0.033	0.012	
*C*_2_	0.524	0.033	0.013		0.520	0.031	0.016	
Δ	0.006	0.010	0.009	5.5	0.002	0.013	0.012	4.2
*β*_1 _= *β*_2 _= *β*_3 _= *log*(2)								
*C*_1_	0.784	0.013	0.006		0.784	0.013	0.006	
*C*_2_	0.881	0.011	0.006		0.866	0.011	0.006	
Δ	0.097	0.005	0.005	100	0.082	0.005	0.004	100

Z_2 _uniformly distributed								
*β*_1 _= *β*_2 _= *β*_3 _= 0								
*C*_1_	0.532	0.055	0.019		0.532	0.055	0.019	
*C*_2_	0.540	0.055	0.019		0.541	0.055	0.020	
Δ	0.008	0.015	0.013	2.2	0.009	0.017	0.013	4.0
*β*_1 _= *β*_2 _= *β*_3 _= *log*(2)								
*C*_1_	0.733	0.029	0.014		0.733	0.029	0.014	
*C*_2_	0.781	0.027	0.012		0.785	0.027	0.012	
Δ	0.048	0.009	0.009	100	0.052	0.010	0.010	100

Results of 1000 simulations

*C*_1_, Harrell's C index of the proportional hazard model without the phase-2 variable

*C*_2_, Harrell's C index of the proportional hazard model with the phase-2 variable

Δ, Harrell's predictive value of the phase-2 variable, *H*0: Δ = 0

Mean of the predictive ability estimates (Est), mean of the standard error estimates $\overset{\wedge }{\mathrm{SE}}$ and standard error of the estimates (SE), with a correctly specified normal imputation model (*Z*_2 _normally distributed), and with two misspecified normal imputation models (*Z*_2 _log-normally and uniformly distributed)

### Application to the Three-City study

The mean fraction of missing information about the effect of D-dimer was 4.9 and 3.7 per cent for CHD and VaD risks, respectively. Table 4 gives the estimated hazard ratios associated with D-dimer tertiles. The MI and the weighted approaches yielded similar estimates and precision. The CI of the hazard ratio associated with the linear effect of a one-tertile difference were respectively (0.94-1.38) versus (0.92-1.38) for CHD and (1.13-2.53) versus (1.13-2.67) for VaD. For phase-1 variables, both estimators provided similar results, but MI was always the more precise (data not shown).

**Table 4:** Estimates of hazard ratios (HR) and 95% confidence interval (CI) associated with D-dimer tertiles.

	Multiple imputation estimates	Weighted estimates
	
	HR (95% CI)	HR (95% CI)
Risk of CHD and D-Dimer^a^		
T1	1.00 (reference)	1.00 (reference)
T2	1.42 (0.99-2.04)	1.40 (0.97-2.04)
T3	1.32 (0.89-1.97)	1.30 (0.84-1.99)
Linear trend	1.14 (0.94-1.38)	1.13 (0.92-1.38)
Risk of VaD and D-Dimer^b^		
T1	1.00 (reference)	1.00 (reference)
T2	1.57 (0.63-3.93)	1.60 (0.63-4.09)
T3	2.77 (1.17-6.57)	2.93 (1.22-7.06)
Linear trend	1.69 (1.13-2.53)	1.74 (1.13-2.67)

CHD, cardiovascular heart disease; T1, tertile 1; T2, tertile 2; T3, tertile 3; VaD, vascular dementia

^a ^Adjusted for age, center, sex, body mass index, hypertension, hypercholesterolemia, diabetes, diabetes drugs, tobacco use

^b ^Adjusted for age, center, sex, educational level, body mass index, apolipoprotein *ε*4

Harrell's *C *for the models including only phase-1 variables were above 0.69 for CHD risk and above 0.86 for VaD risk (Table 5). Hence, CHD and VaD risks were largely explained by standard risk factors, and the inclusion of plasma D-dimer levels did not significantly improve the predictive ability of the model, despite the fact that elevated D-dimer levels significantly increased the VaD risk. For CHD as for VaD, the index did not significantly differ from 0.

**Table 5:** Predictive ability and 95% confidence interval (CI) of D-Dimer tertiles on cardiovascular heart disease (CHD) and vascular dementia (VaD) risks.

		CHD		VaD
	
	Estimate	95% CI	Estimate	95% CI
*C*_1_	0.693	(0.622-0.764)	0.865	(0.787-0.943)
*C*_2_	0.694	(0.621-0.767)	0.874	(0.798-0.950)
Δ	0.002	(-0.004-0.008)	0.009	(-0.011-0.029)
NRI	0.009	(-0.049-0.066)	-	-
IDI	0.001	(-0.001-0.003)	0.0004	(-0.0002-0.0010)

*C*_1_, Harrell's C index of the proportional hazard model without the phase-2 variable

*C*_2_, Harrell's C index of the proportional hazard model with the phase-2 variable

Δ, Harrell's predictive ability of the phase-2 variable

NRI, net reclassification improvement by adding the phase-2 variable IDI, integrated discrimination index by adding the phase-2 variable

## Discussion

Use of a consistent estimator does not guarantee the absence of any bias for finite sample. We only showed that MI analysis of case-cohort data provides unbiased estimates of the log-hazard ratio when the imputation model and the proportional hazard model are correctly specified. The misspecification of the imputation model can originate from an erroneous choice of the distribution, or from wrongly assuming that the estimator of the imputation model is consistent and normal, or from the omission of some important explanatory variable. Imputations carried out using a misspecified distribution in the imputation model can provide biased estimates of hazard ratios, especially, if the specified distribution of the phase-2 variable differs from the true one in terms of symmetry (log-normal versus normal distribution). The negative bias on a log hazard ratio of 0.69 was noticeable but not large when a log-normal variable was imputed according to a normal distribution (-0.09 or -13%), but it is clearly a type of misspecification easily identified with diagnostic tools [20]. One can then transform the incomplete variable in order to obtain a symmetrical distribution, impute transformed values and apply the inverse transformation to the imputed values. Note that although a normal and a uniform distribution are quite different, both are symmetrical and the observed bias was quite smaller (only 5%). In the 3C study of the relationship between VaD and D-dimer, we observed slightly different estimates of the log hazard ratio when comparing the third to the first tertile (2.77 versus 2.93, i.e. a relative difference of 8% between the MI and the weighted estimates). This is probably because of the qualitative imputation of D-dimer, and thus, the use of a multinomial imputation model, which implied estimation of parameters in separate strata defined by D-dimer concentration tertiles, some of which had a small number of events. Due to these small numbers (only 51 VaD in total), asymptotic conditions might not have been fulfilled in at least some strata, and the estimated coefficients of the imputation model could have been biased and notnormally distributed. We give below some recommendations regarding the choice of explanatory variables in the imputation model. Since the potential bias of MI estimates can be detected by comparing them to weighted estimates, we suggest building the proportional hazard model by using only the case-cohort data and weighted estimators. MI can eventually be used to reanalyze the data with the selected model to improve the precision of the results, while verifying that no bias was introduced.

In simulated data, for the phase-1 variables, the precision of MI and full cohort estimates was similar and smaller than with the weighted estimator. For the phase-2 variable, MI estimates were slightly more precise than weighted estimates. Globally, the mean squared errors were smaller with MI than with the weighted estimator, with one exception implying a normal imputation model for a log-normally distributed phase-2 variable, an error which should easily be avoided.

There is no standard method for estimating the predictive ability of a model in the framework of case-cohort surveys. We showed that the naive application of the *C *index to case-cohort surveys yielded an underestimation of the predictive ability of the model that depended on the subcohort size when the phase-2 variable had an effect on the risk. Similarly, the naive estimates of the predictive ability of an added phase-2 variable differed notably from the full cohort values when the effect of the phase-2 variable was not null. Harrell's C index could theoretically be estimated with a weighted approach, but this can be computationally difficult because it requires weighting each pair by the pairwise sampling probabilities, i.e., using a square matrix of size *N'*(*N'*-1), where *N' *is the size of the case-cohort sample. Computing the variance of this Horvitz-Thompson estimator requires either weighting each quadruplet by the quadruple-wise sampling probabilities, i.e., working with a matrix of size *N*'(*N*'-1)(*N*'-2)(*N*'-3), or bootstrapping the case-cohort data. By contrast, MI easily allows estimation of the predictive ability of a model or of an additional phase-2 variable and their variances in the context of case-cohort data, only requiring bootstrapping to estimate the variance of the predictive ability of the phase-2 variable. MI provided estimates of Harrell *C*, NRI and IDI indexes similar to those obtained with the full cohort analysis. Note, however, that the predictive abilities were always overestimated because the same data were used to estimate the model and its predictive ability.

Analysis of the Three-City case-cohort study was in agreement with our previous work [10]. The weighted and the MI approaches yielded similar estimates of the hazard ratios and MI was slightly more precise, particularly for phase-1 variables. The relative differences between both estimates was always below 2% for the hazard ratios related to CHD and D-dimer, but as early discussed, they could be slightly higher (8%) for a hazard ratio related to VaD and D-dimer. The precision was similar for both analyses.

The imputation model must reflect the association between the incomplete variable, the outcome and the other explanatory variables. Therefore, variables included in the proportional hazard model as well as the stratification variables must be included in the imputation model. If a surrogate of the phase-2 variable is available, it should also be included in the imputation model. On the other hand, multiple imputation approach can provide unbiased and more efficient estimates than weighted analysis even when no strong predictor of the phase-2 variable is available [10]. The inclusion of additional variables other than strongly predictive variables can lead to an increased inter-imputation variance. This prompted the use of different imputation models for D-dimer levels in the CHD and VaD analyses. However, we verified that adding the variables only used in the CHD analysis to the model used for VaD, did not modify the results observed in the former (data not shown).

The number of requested imputations depends on the proportion of missing information which, in case-cohort studies, is considerably smaller than the percentage of incompletely observed subjects. Rubin showed that with as much as 40 per cent information missing, M = 5 imputations provides an asymptotic relative efficiency was 0.97, and, with 50 per cent missing information, M = 10 provides an asymptotic relative efficiency of 0.98. Thus, a small number of imputations, 5-10, should suffice [21]. In our analyses, we used 5 imputations to limit the computer time of the simulations, a reasonable choice since the proportion of missing information was always smaller than 30 per cent. However, a slightly larger number of imputations (e.g. 10) could have been performed on the 3C study data at a reasonable time cost; it would have provided a more precise estimate of the between imputation variance and of the percentage of missing information.

The VaD risk increased with D-dimer tertiles. However, D-dimer inclusion did not significantly improve the predictive ability of the model for VaD risk. Computations of the *C *and IDI index yielded the same conclusion. To our knowledge, no other results concerning the predictive ability of D-dimer on the risk of VaD have been published to date. The risk of CHD did not vary with D-dimer, so, not surprisingly, the predictive ability of this variable was negligible, regardless of the index used. Wang *et al. *[22] and Tzoulaki [23] reported that the use of 10 and 4 biomarkers respectively added only moderately to the overall risk prediction based on conventional cardiovascular risk factors.

## Conclusions

MI is a simple alternative approach to weighted analysis for analyzing case-cohort surveys, obtaining correct estimates of the log hazard ratios and their standard errors, improving precision for the phase-1 variable estimates, and providing at least the same precision as weighted estimators for phase-2 variable estimates. It allows an easy evaluation of the predictive ability of the model and, more generally, any tool proposed in the framework of cohort studies can be applied to case-cohort data using MI.

## Abbreviations

MI: Multiple imputation; CHD: Coronary heart disease; VaD: Vascular dementia; NRI: Net reclassification index; IDI: Integrated discrimination index.

## Competing interests

The authors declare that they have no competing interests.

## Authors' contributions

HM conducted the literature review, simulations, data analyses and wrote the manuscript. LC conducted the analysis of the relationship between D-dimer levels and CHD and VaD risks and supervised the epidemiological aspects of the application to the Three-City study. MC conducted and supervised the writing of the manuscript. All authors have read the manuscript, are in agreement that the work is ready for submission to the journal, and accept responsibility for the manuscript's contents.

## Pre-publication history

The pre-publication history for this paper can be accessed here:

http://www.biomedcentral.com/1471-2288/12/24/prepub
